# A novel protein encoded by circTUBGCP3 blocks ferroptosis and promotes gastric cancer progression

**DOI:** 10.1016/j.jbc.2025.110507

**Published:** 2025-07-21

**Authors:** Shanshan Chang, Xianli Fu, Longquan Xiang, Yuqian Yuan, Siyu Xiao, Lehua Peng, Xiaoya Xie, Xinyi Kang, Xianling Feng, Hassan Ashktorab, Yin Peng, Zhe Jin, Xiaojing Zhang

**Affiliations:** 1 Guangdong Provincial Key Laboratory of Genome Stability and Disease Prevention, Department of Pathology, Shenzhen University Medical School, Shenzhen University, Shenzhen, Guangdong, People's Republic of China; 2 ShenZhen University General Hospita, Shenzhen University, Shenzhen, Guangdong, People's Republic of China; 3 Department of Pathology, Jining No.1 People's Hospital, Jining, Shandong, People's Republic of China; 4 Department of Medicine and Cancer Center, Howard University, College of Medicine, Washington, District of Columbia, USA

**Keywords:** circTUBGCP3, TUBGCP3-230aa, gastric cancer, ferroptosis, novel protein

## Abstract

Circular RNAs play significant roles in the development and progression of various cancers through diverse mechanisms, including the translation of novel proteins. Ferroptosis, a recently identified form of cell death, is associated with tumorigenesis in several cancers; however, its pathological mechanisms in gastric cancer (GC) remain unclear. Here, we found that circTUBGCP3 expression was elevated in GC tissues compared with normal gastric tissues. Moreover, circTUBGCP3 can be translated into a previously undescribed protein, TUBGCP3-230aa. *In vitro* and *in vivo* functional analyses demonstrated that both circTUBGCP3 and TUBGCP3-230aa promote rapid GC cell proliferation, with TUBGCP3-230aa exerting independent biological effects. Enolase 1 (ENO1), a glycolytic enzyme, was identified as an interacting partner of TUBGCP3-230aa, leading to activation of the glycolytic pathway and inhibition of ferroptosis in GC cells in *vitro* and *in vivo.* Mechanistically, TUBGCP3-230aa stabilizes ENO1 through posttranslational regulation, thereby repressing ferroptosis. Together, our results identify circTUBGCP3 and TUBGCP3-230aa as potential biomarkers for GC and uncover a novel mechanism of ferroptosis regulation, which may represent a promising therapeutic target. Furthermore, our findings highlight a critical moonlighting function of ENO1 in GC and underscore its potential as a novel target for cancer therapy.

Gastric cancer (GC) is a highly heterogeneous tumor and the third leading cause of cancer-related death worldwide (1, 2, 3). Due to its rapid progression and the absence of early diagnostic indicators, GC is typically diagnosed at an advanced stage. Even after radical resection combined with chemoradiotherapy, recurrence and treatment resistance are common, and the prognosis remains poor (4). Therefore, there is an urgent need to further investigate the molecular pathology of GC and identify novel therapeutic strategies.

Metabolic reprogramming is a hallmark of cancer and is most commonly manifested as abnormal aerobic glycolysis (5, 6). Even under normoxic conditions, cancer cells often rely on glycolysis to meet their increased energy and biosynthetic demands-a phenomenon known as the Warburg effect (7, 8). Ferroptosis, a recently characterized, iron-dependent form of regulated cell death, is tightly linked to metabolic pathways. It is driven by excessive lipid peroxidation and is biochemically and genetically distinct from autophagy, apoptosis, and pyroptosis (9, 10). While ferroptosis has been implicated in various cancers and is regulated by multiple oncogenic signaling pathways (11, 12), its role and regulatory mechanisms in GC remain poorly understood.

One promising area of investigation involves circular RNAs (circRNAs)—a class of covalently closed RNA transcripts lacking 5′-3′ polarity and polyadenylated tails (13, 14). Although traditionally considered non-coding RNAs that function as miRNA sponges or protein scaffolds (13, 15), recent studies have shown that some circRNAs can encode functional proteins (16). These circRNA-encoded proteins have been shown to regulate cell proliferation, migration, invasion, and cell death and have been implicated in the development of multiple tumor types, including GC (17, 18, 19, 20). Given their unique expression profiles, such proteins may serve as novel diagnostic markers or therapeutic targets in GC.

In this study, we aimed to identify translatable circRNAs differentially expressed in GC. We found that one such circRNA, circTUBGCP3, was highly expressed in GC tissues and had protein-coding potential. We subsequently investigated the function of the protein encoded by circTUBGCP3, focusing on its protein-protein interactions and its effects on cell growth, metabolism, and ferroptosis *in vitro*, as well as its role in tumorigenicity *in vivo*.

## Results

### Circular TUBGCP3 RNA is differentially expressed in GC

**Figure 1:**
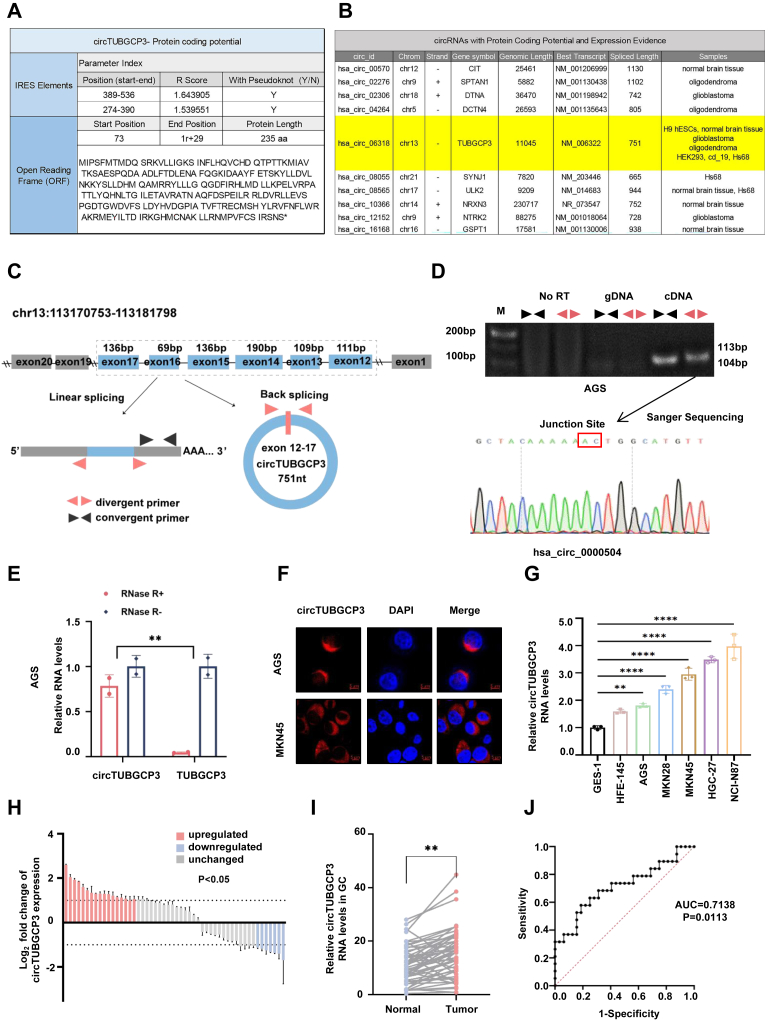
**CircTUBGCP3 is highly expressed in GC.***A*, prediction of the potential coding circRNAs by circRNADb. *B*, CircRNAs with coding potential and expression evidence. *C*, illustration of the annotated genomic region of TUBGCP3 and putative RNA splicing forms. Convergent and divergent primers were designed to amplify linear and back-spliced products. *D*, PCR analyses of cDNA and gDNA extracted from AGS cells were performed using divergent and convergent primers to examine circTUBGCP3 and linear TUBGCP3 mRNA. Subsequent Sanger sequencing identified the junction sequence of circTUBGCP3 in AGS cells. *E*, RT-qPCR to detect the expression of circTUBGCP3 in AGS cells following RNase R treatment. *F*, FISH was performed to identify the subcellular localization of circTUBGCP3. FISH probes were designed according to the circTUBGCP3 junction site, and circTUBGCP3 overexpression plasmids were used independently in AGS and MKN45 cells to verify the specificity of these probes. Scale bar, 5 μm. *G*, relative circTUBGCP3 RNA levels in gastric cells. *H*, Fold changes in circTUBGCP3 expression in tumor specimens and paired adjacent gastric tissues in a cohort of GC patients (n = 52). *I*, relative circTUBGCP3 expression levels in the same cohort (n = 52); two-sided paired *t* test, *p* < 0.005. *J*, receiver–operating characteristic (ROC) curve analysis of normalized circTUBGCP3 expression in GC with and without lymph node metastasis. The area under the ROC curve (AUC) conveys the accuracy, in terms of sensitivity and specificity, of this biomarker in distinguishing GC lymph node metastasis. These data are pooled from three independent experiments and are presented as mean ± SD. Where applicable, the unpaired two-tailed Student’s *t* test was used to determine the significance of differences between the indicated groups; ∗*p* < 0.05; ∗∗*p* < 0.01; ∗∗∗*p* < 0.001.

In this study, we aimed to identify potentially translatable circRNAs in gastric cancer (GC). Using CircRNADb, a comprehensive database of human circular RNAs with predicted coding potential, we identified circTUBGCP3 as one such candidate (Fig. 1*A*). This database also showed that circTUBGCP3 is detectable in a variety of human tissues and cell lines, including normal brain tissue and glioblastoma (Fig. 1*B*). Additionally, circTUBGCP3 was among the differentially expressed circRNAs identified in our previous RNA sequencing analysis of GC tissues (21). Focusing on circRNAs relevant to GC, we annotated the identified candidates using circBase. As shown in Figure 1*C*, circTUBGCP3 is located on chromosome 13q34 and is generated by back-splicing exons 12 to 17 of the *TUBGCP3* gene, resulting in a 751-nt transcript. Junction-specific divergent and convergent primers were designed to amplify circTUBGCP3 (Fig. 1*C*). To confirm back-splicing, we used these primers to selectively amplify circTUBGCP3 from cDNA rather than genomic DNA (gDNA) (Fig. 1*D*, upper panel). The predicted backsplice junction was validated by Sanger sequencing and found to match the circBase entry (ID: hsa_circ_0000504) (Fig. 1*D*, lower panel), confirming the presence of endogenous circTUBGCP3 in GC. RNase R treatment reduced levels of linear TUBGCP3 mRNA but not circTUBGCP3, indicating the circular RNA’s high stability (Fig. 1*E*).

Next, to determine circTUBGCP3 localization, we performed FISH in MKN45 and AGS cells using a junction-specific probe for circTUBGCP3. We found that circTUBGCP3 was mainly localized in the cytoplasm of these cells (Fig. 1*F*). This finding was further validated by RT-qPCR analysis of endogenous GC cell fractions (Fig. S1). We also compared the expression levels of circTUBGCP3 in GC cell lines (HGC27, AGS, MKN45, NCI-N87, and MKN28) and immortalized gastric epithelial cells (HFE145 and GES1) using junction-specific RT-qPCR primers. The results showed that circTUBGCP3 expression was significantly higher in GC cells than in non-tumorigenic epithelial cells (Fig. 1*G*). To explore potential clinical implications, we analyzed circTUBGCP3 expression in 52 paired GC and adjacent normal tissue samples by RT-qPCR. circTUBGCP3 expression was elevated in 61.5% (32/52) of GC samples compared to their matched normal tissues (Fig. 1, *H* and *I*). In 17 of these samples, expression was more than 2-fold higher in tumor tissue. Receiver operating characteristic (ROC) curve analysis based on circTUBGCP3 expression levels in GC with *versus* without lymph node metastasis yielded an area under the curve (AUC) of 0.7138 (*p* = 0.0113) (Fig. 1*J*). Correlation analyses revealed that circTUBGCP3 expression was significantly higher in poorly differentiated tumors compared to well- to poorly differentiated tumors (*p* = 0.0037), and in T3–T4 tumors compared to T1–T2 tumors (*p* = 0.0027), indicating a possible link with tumor invasiveness. Additionally, ROC curve analysis showed that circTUBGCP3 expression levels correlated with lymph node metastasis, with circTUBGCP3 particularly highly expressed in GCs with lymph node metastases (*p* = 0.0164) (Table 1). These findings suggest that circTUBGCP3 may serve as a promising diagnostic and prognostic biomarker in GC.

**Table 1:** Correlation analysis between the expression of circTUBGCP3 and the clinicopathological indices

Features	N	High expression	Low expression	*p* Value
Total cases	50	30	20	
Sex				
Male	36	24	12	**0.3385**
Female	14	7	7	
Tumor size				
<5 cm	25	12	13	**0.0889**
>5 cm	26	19	7	
Differentiation				
Well-poor	22	8	14	**0.0037**
poor	28	22	6	
Lymphatic metastasis				
Yes	31	23	8	**0.0164**
No	19	7	12	
Tumor stage				
T1+T2	13	3	10	**0.0027**
T3+T4	37	27	10	

Bold values are statistically significant *p* < 0.05.

### CircTUBGCP3 encodes the novel protein TUBGCP3-230aa

**Figure 2:**
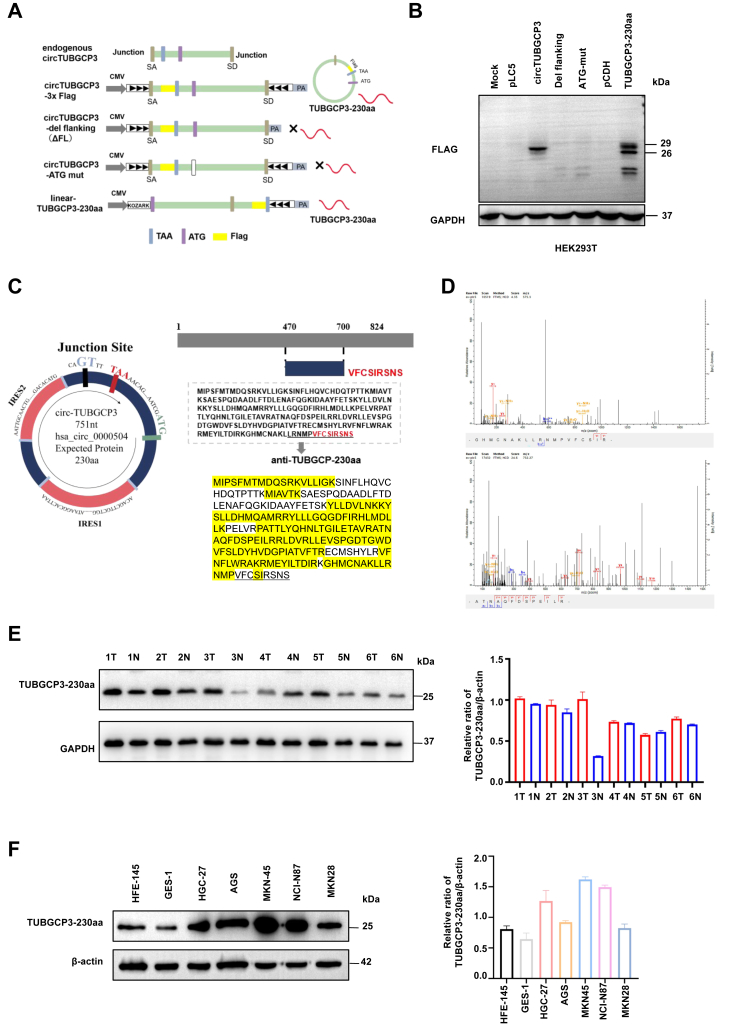
**CircTUBGCP3 encodes the peptide TUBGCP3-230aa.***A*, illustration of endogenous circTUBGCP3, the circTUBGCP3 construct, the del-flanking construct, ATG mut construct, and the linearized TUBGCP3-230aa construct. *B*, immunoblot of cells overexpressing the above constructs using custom anti-FLAG antibody to detect expression of TUBGCP3-230aa. *C*, *Left panel*: Schematic representation of the circTUBGCP3 open reading frame (ORF), including the junction site. The circTUBGCP3-encoded peptide, TUBGCP3-230aa, contains a unique C-terminus generated by an ORF spanning more than 360° of the circular transcript. Right panel: Schematic of the TUBGCP3-230aa sequence. The protein shares homology with the central region of full-length TUBGCP3, while the 9 unique C-terminal residues are shown in *red*. A custom antibody was generated against the underlined unique C-terminal peptide. Shared peptides are shown in *black*; peptides identified by IP-MS are highlighted in *yellow*; the unique C-terminal peptide is underlined. *D*, IP assay to detect endogenous TUBGCP3-230aa using anti-FLAG antibody. IP/MS was performed on 15 to 35 kDa proteins excised from SDS-PAGE gels. *Upper panel*: LC/MS spectra showing peptides from the unique C-terminal region; *lower panel*: LC/MS spectra of peptides homologous to the parent protein. *E*, *Left* panel: Immunoblot detecting TUBGCP3-230aa in six randomly selected paired GC samples using the anti-TUBGCP3-230aa antibody. *Right panel*: Semiquantitative analysis of TUBGCP3-230aa expression based on greyscale intensity in GC samples. N: normal tissues; T: tumor tissues. *F*, *Left* panel: TUBGCP3-230aa expression levels in gastric cell lines determined by immunoblotting. *Right* panel: Semiquantitative greyscale analysis of TUBGCP3-230aa protein levels in GC cell lines. All data were pooled from three independent experiments and are presented as mean ± SD; two-sided paired *t* test; ∗*p* < 0.05; ∗∗*p* < 0.01; ∗∗∗*p* < 0.001. N: normal; T: tumor.

Recent studies have reported that certain circRNAs can encode functional proteins in cancer (8, 11, 12, 13). We previously showed that ORFs spanning more than 360 degrees in circRNAs can generate unique molecular targets in cancer. In circTUBGCP3, we identified a similar ORF predicted to encode a novel protein, which we named TUBGCP3-230aa. As with most translatable circRNAs, translation of this ORF was predicted to be driven by IRESs. Sequence analysis of circTUBGCP3 revealed two putative IRES elements, as predicted using the IRESite database (Fig. S2, upper panel). To test their activity, we cloned wild-type and mutant IRES sequences into dual luciferase reporter plasmids and transfected them into 293T cells. Luciferase assays confirmed that the IRES spanning nucleotides 606 to 721 exhibited strong translational activity (Fig. S2, lower panel). To validate the coding potential of circTUBGCP3, we constructed a circTUBGCP3 overexpression vector containing a 3 × FLAG tag inserted just before the predicted stop codon of the ORF. Immunoblotting using an anti-FLAG antibody detected expression of a FLAG-tagged protein in 293T cells transfected with the circTUBGCP3-3 × FLAG construct. A linear TUBGCP3-230aa-3 × FLAG overexpression vector served as a positive control. By contrast, FLAG expression was absent when the start codon was mutated or the flanking sequences necessary for circularization were deleted (Fig. 2, *A* and *B*). Empty pLC5 and pCDH vectors served as negative controls. Together, these results demonstrated that circTUBGCP3 contains a cross-junction ORF capable of encoding a novel protein, TUBGCP3-230aa (Fig. 2*B*).

TUBGCP3-230aa shares its N-terminal sequence with TUBGCP3, corresponding to amino acids 470 to 700, but contains a unique C-terminal region generated by a frameshift during the second round of translation. This adds nine amino acids not present in the parent protein (Fig. 2*C*). To confirm the endogenous expression of TUBGCP3-230aa in GC cells, we performed IP followed by SDS-PAGE on MKN45 cell lysates. Protein bands between 15 and 35 kDa were excised and subjected to liquid chromatography–mass spectrometry (LC-MS). LC-MS analysis identified peptides corresponding to the unique C-terminal sequence of TUBGCP3-230aa (Fig. 2*D*, upper panel) as well as peptides shared with the parental TUBGCP3 protein (Fig. 2*D*, lower panel). We then generated a custom antibody against the unique C-terminal sequence (VFCSIRSNS) of human TUBGCP3-230aa (Fig. 2*C*) and used it to detect endogenous expression of the novel protein in GC tissues and cell lines. Immunoblotting showed that TUBGCP3-230aa was significantly upregulated in 5 out of 6 randomly selected GC tissue samples compared with matched adjacent normal tissues (Fig. 2*E*). Furthermore, TUBGCP3-230aa protein expression was markedly higher in GC cell lines (HGC27, AGS, MKN28, NCI-N87, and MKN45) than in non-cancerous gastric epithelial cells (HFE145 and GES1) (Fig. 2*F*).

### The tumorigenic function of circTUBGCP3 and TUBGCP3-230aa in GC *in vitro* and *in vivo*

**Figure 3:**
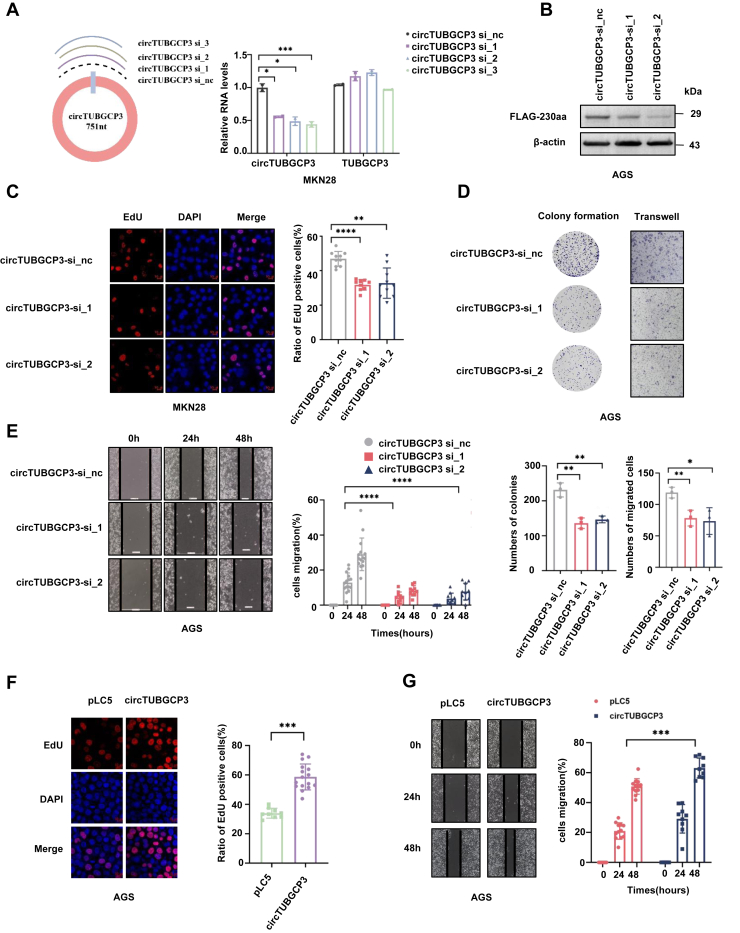
**Biological functions of circTUBGCP3 in GC cell lines.***A*, illustration of the circTUBGCP3 junction-specific siRNA target site. *B*, GC cells were transfected with circTUBGCP3 siRNA-1/2/3 or a negative control (nc) siRNA, and circTUBGCP3 expression was analyzed by RT-qPCR and immunoblotting. *C–G*, cell proliferation, migration, and colony formation were measured by EdU (*C* and *F*), colony formation (*D*, *left* panel), transwell (*D*, *right* panel), and scratch (*E* and *G*) assays in cells with circTUBGCP3 overexpression or knockdown using circTUBGCP3 siRNAs. The data were pooled from three independent experiments and are presented as mean ± SD. Where applicable, the unpaired two-tailed Student’s *t* test was used to determine the significance of differences between the indicated groups; ∗*p* < 0.05; ∗∗*p* < 0.01; ∗∗∗*p* < 0.001.

**Figure 4:**
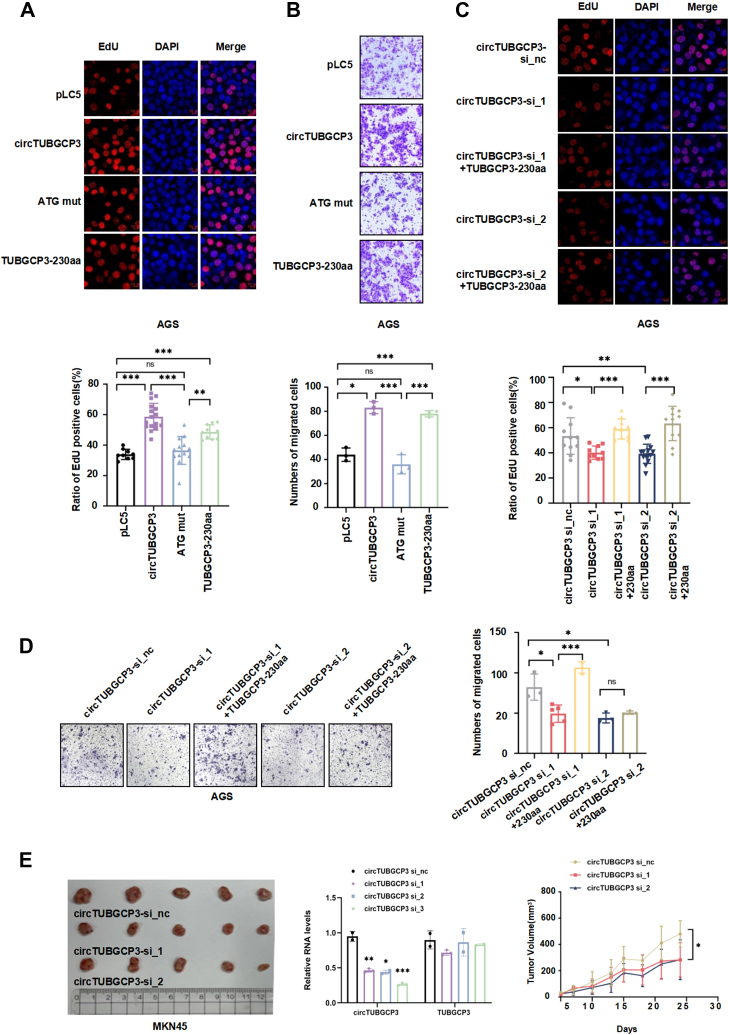
**The independent biological functions of TUBGCP3-230aa in GC *in vitro* and *in vivo*.** Cell proliferation and migration were measured by EdU and transwell assays, respectively. EdU (*A* and *C*) and transwell (*B* and *D*) assays were carried out using AGS cells transfected with circTUBGCP3 constructs or circTUBGCP3 siRNAs. Rescue assays were performed by co-transfecting AGS cells with circTUBGCP3 siRNAs and TUBGCP3-230aa. *E*, subcutaneous tumor xenograft assays. The *left* panel shows tumor volumes from each group under different treatments. The *right* panel shows the interference efficiency of circTUBGCP3 siRNAs in tumor tissues was analyzed by RT-qPCR. The data were pooled from three independent experiments and are presented as mean ± SD. Where applicable, the unpaired two-tailed Student’s *t* test was used to determine the significance of differences between the indicated groups; ∗*p* < 0.05; ∗∗*p* < 0.01; ∗∗∗*p* < 0.001.

To investigate the role of circTUBGCP3 in GC, we performed gain- and loss-of-function experiments in GC cell lines. First, three siRNAs targeting the specific back splice junction of circTUBGCP3 were designed (Fig. 3*A*, left panel). Transfection of these siRNAs significantly reduced circTUBGCP3 levels in MKN28 cells, while the expression of linear TUBGCP3 remained largely unchanged (Fig. 3*A*, right panel). In AGS cells, siRNA-mediated knockdown also led to a marked decrease in TUBGCP3-230aa protein levels (Fig. 3*B*). Functional assays demonstrated that knockdown of circTUBGCP3 significantly suppressed cell proliferation and colony-forming ability, as shown by EdU and colony formation assays in both MKN28 and AGS cells (Fig. 3, *C* and *D*, left panel). In addition, transwell and wound-healing assays showed a significant reduction in AGS cell migration upon circTUBGCP3 knockdown (Fig. 3*D*, right panel; Fig. 3*E*). Conversely, overexpression of circTUBGCP3 in AGS cells significantly increased both proliferation and migration compared with controls, as shown by EdU and wound-healing assays (Fig. 3, *F* and *G*). To explore the independent role of TUBGCP3-230aa, we transfected AGS cells with either the linear TUBGCP3-230aa overexpression construct or co-transfected them with circTUBGCP3 siRNAs and TUBGCP3-230aa. EdU assays revealed that AGS cells transfected with either circTUBGCP3 or TUBGCP3-230aa exhibited significantly enhanced proliferation compared with cells transfected with pLC5 or the ATG mutant control (Fig. 4*A*). Similarly, wound-healing and transwell assays showed that cell motility was significantly increased upon overexpression of either circTUBGCP3 or TUBGCP3-230aa (Figs. 4*B* and S3). Finally, rescue assays were performed by co-transfecting AGS cells with circTUBGCP3 siRNAs and TUBGCP3-230aa. Knockdown of circTUBGCP3 by circTUBGCP3 siRNAs in AGS cells significantly attenuated cell proliferation and migration abilities, while co-expression of circTUBGCP3 siRNAs and TUBGCP3-230aa reversed the effects of the circTUBGCP3 siRNAs on cell proliferation and migration (Fig. 4, *C* and *D*).

To assess the oncogenic role of circTUBGCP3 *in vivo*, MKN45 cells were injected subcutaneously into nude mice to establish xenograft models. Mice were treated with junction-specific circTUBGCP3 siRNAs or control siRNA *via* peritumoral injection twice a week for 3 weeks. Knockdown of circTUBGCP3 significantly reduced tumor volume and inhibited tumor growth *in vivo* (Fig. 4*E*, left panel). RT-qPCR confirmed efficient silencing of circTUBGCP3 in the tumors (Fig. 4*E*, right panel). Taken together, these results demonstrate that circTUBGCP3 functions as an oncogene in GC by promoting proliferation and migration both *in vitro* and *in vivo.* Furthermore, TUBGCP3-230aa is a novel protein with independent tumor-promoting activity in GC.

### TUBGCP3-230aa binds to ENO1 protein and regulates its stability

**Figure 5:**
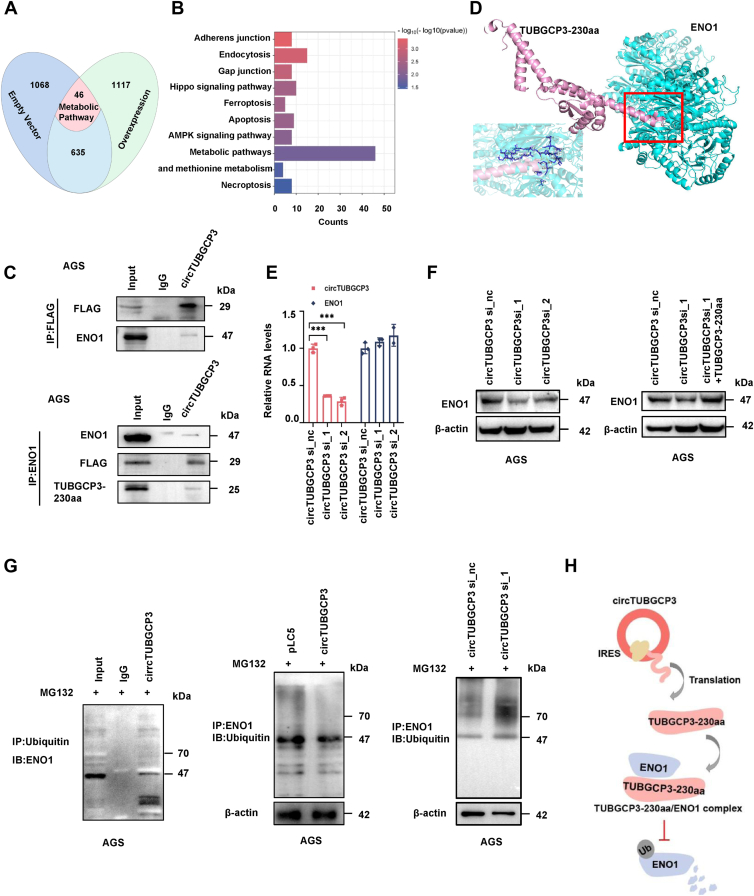
**TUBGCP3-230aa interacts with ENO1 and regulates ENO1 stability.***A*, Immunoprecipitation–mass spectrometry (IP–MS) was conducted on AGS cells transfected with FLAG-tagged circTUBGCP3 or an empty vector. *B*, KEGG analysis of the proteins (identified by MS) interacting with TUBGCP3-230aa in GC cells. The results indicate the involvement of TUBGCP3-230aa in metabolic pathways such as glycolysis and ferroptosis. *C*, Co-IP assays showed an interaction between TUBGCP3-230aa and ENO1. *D*, ClusPro 2.0 was used to simulate binding models of TUBGCP3-230aa or linear TUBGCP3 with ENO1, and PyMOL software was used for visualization and analysis. *E*, RT-qPCR analysis of ENO1 mRNA levels following circTUBGCP3 knockdown. *F*, immunoblotting to assess ENO1 protein levels regulated by TUBGCP3-230aa (*left* panel), and rescue assays showing that co-transfection of circTUBGCP3 siRNAs and linear TUBGCP3-230aa restored ENO1 levels (*right* panel). *G*, Co-IP assay showing IP of ENO1 with anti-ubiquitin antibody (*left* panel); immunoblot detection of ENO1 ubiquitination in MG132-treated cells with or without circTUBGCP3 overexpression (*middle* panel); and ENO1 ubiquitination in MG132-treated cells with circTUBGCP3 knockdown (*right* panel). *H*, schematic diagram illustrating how TUBGCP3-230aa protects ENO1 stability.

To explore the molecular mechanism underlying the function of TUBGCP3-230aa, we examined its potential protein–protein interaction partners by conducting IP–MS. The IP was conducted using an anti-FLAG antibody in AGS cells transfected with either FLAG-tagged circTUBGCP3 or the empty vector (pLC5). IP–MS analysis identified 1068 proteins in pLC5-transfected cells and 1117 in circTUBGCP3-overexpressing cells, with 635 shared proteins. Among these, 46 were associated with metabolic pathways (Fig. 5*A*). KEGG pathway enrichment analysis of the differentially expressed proteins revealed enrichment in pathways such as glycolysis and ferroptosis in TUBGCP3-230aa-expressing cells (Fig. 5*B*). Further analysis of the IP–MS data revealed higher levels of the glycolytic enzyme ENO1 in the circTUBGCP3-overexpressing group compared with the control, suggesting that ENO1 may interact with TUBGCP3-230aa. ENO1, a well-established glycolytic enzyme, is widely recognized as a tumor marker and a potential therapeutic target (22). To confirm the interaction between TUBGCP3-230aa and ENO1, we carried out Co-IP assays. FLAG-tagged circTUBGCP3 was overexpressed in AGS cells and immunoprecipitated using an anti-FLAG antibody. Immunoblotting with anti-ENO1 confirmed the presence of ENO1 in the FLAG-immunoprecipitated complex (Fig. 5*C*, upper panel). Reciprocally, TUBGCP3-230aa was detected by both anti-FLAG and anti-TUBGCP3-230aa antibodies following IP with anti-ENO1 (Fig. 5*C*, lower panel), confirming that TUBGCP3-230aa and ENO1 form a protein complex in GC cells. To further explore the interaction mode, we used PyMOL to predict the binding structure between the TUBGCP3-230aa and ENO1. It displayed that the TUBGCP3-230aa sequence is homologous to residues 470 to 700 of the full-length TUBGCP3 precursor protein (907aa total). TUBGCP3-230aa protein's three-dimensional structure modeled by SwissModel, protein–protein docking simulations were performed with ClusPro. The results indicate that: the C-terminal region of TUBGCP3-230aa binds to residues 410 to 430 of ENO1 (434aa total);The 860 to 870 region of the full-length TUBGCP3 precursor (907aa total) interacts with residues 120 to 130 of ENO1.

ClusPro 2.0 was used to simulate docking models of ENO1 with TUBGCP3-230aa and with the parental TUBGCP3 protein. The models were visualized using PyMOL software. Structural analysis showed that the binding sites of TUBGCP3-230aa and parental TUBGCP3 on ENO1 were distinct (Figs. 5*D* and S4). We next investigated the regulatory effect of TUBGCP3-230aa on ENO1 expression. RT-qPCR revealed that ENO1 mRNA levels were unaffected by circTUBGCP3 knockdown in AGS cells (Fig. 5*E*). Western blot analysis, however, showed that ENO1 protein levels were significantly reduced following circTUBGCP3 knockdown, and this reduction was rescued by co-transfection with TUBGCP3-230aa (Fig. 5*F*), suggesting that TUBGCP3-230aa regulates ENO1 post-translationally. As ENO1 is known to undergo degradation *via* the ubiquitin–proteasome pathway (23), we investigated whether TUBGCP3-230aa stabilizes ENO1 by inhibiting its ubiquitination. ENO1 was immunoprecipitated from MG132-treated AGS cells using an anti-ubiquitin antibody. ENO1 was detectable in the immunoprecipitates, confirming its ubiquitination (Fig. 5*G*, left panel). Overexpression of circTUBGCP3 reduced ENO1 ubiquitination (Fig. 5*G*, middle panel), whereas circTUBGCP3 knockdown led to increased ENO1 ubiquitination (Fig. 5*G*, right panel). These findings demonstrate that TUBGCP3-230aa stabilizes ENO1 by inhibiting its ubiquitination (Fig. 5*H*).

### TUBGCP3-230aa binding ENO1 activates the glycolysis pathway in GC

**Figure 6:**
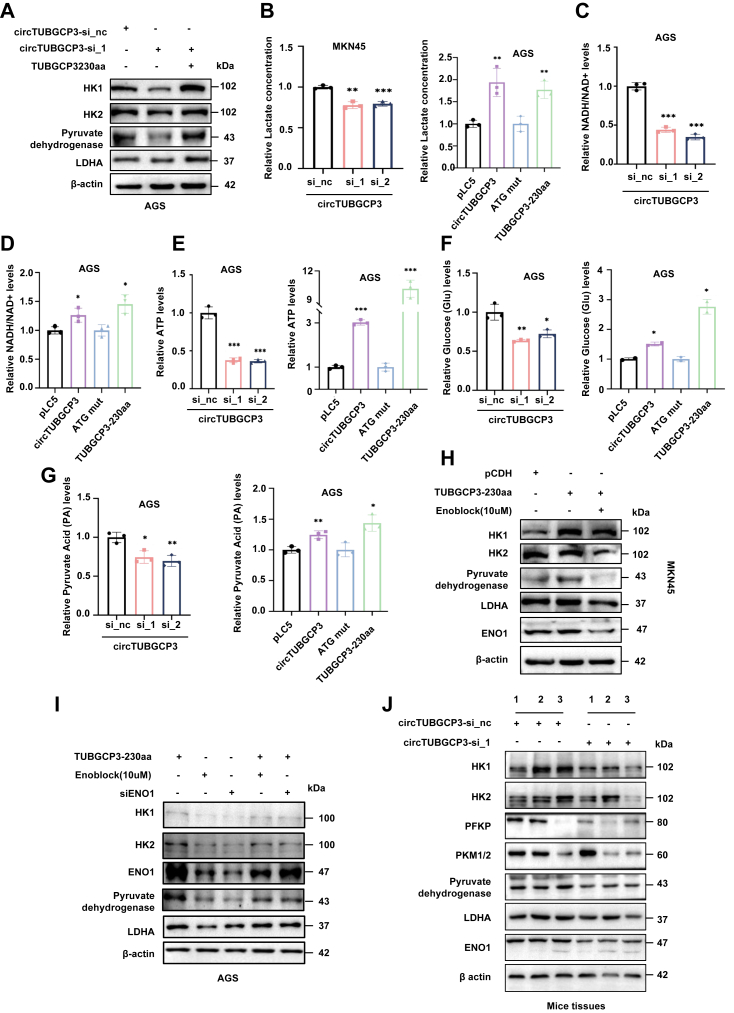
**TUBGCP3-230aa/ENO1 complex activates the glycolysis pathway in GC cells.***A*, Immunoblot to detect key glycolytic enzymes in AGS cells transfected with circTUBGCP3 siRNAs or co-transfected with circTUBGCP3 siRNAs and linear TUBGCP3-230aa. *B*–*G*, detection of lactic acid (*B*), NADH (*C* and *D*), ATP (*E*), glucose (*F*), and pyruvate (*G*) levels in GC cells following circTUBGCP3 knockdown or circTUBGCP3/TUBGCP3-230aa overexpression. *H*, rescue assays detecting glycolytic enzymes in cells treated with linear TUBGCP3-230aa and the ENO1 inhibitor ENOblock. *I*, rescue assays assessing expression of glycolytic enzymes in AGS cells treated with siENO1, ENOblock, or co-transfected with linear TUBGCP3-230aa following ENO1 inhibition. *J*, protein levels of key glycolytic enzymes in tumor tissues from nude mice treated with circTUBGCP3 siRNAs.

Given the known regulatory role of ENO1 in glycolysis, we hypothesized that TUBGCP3-230aa may modulate glycolytic activity in GC cells through its interaction with ENO1. To test this, we evaluated the metabolic consequences of TUBGCP3-230aa expression. In AGS cells transfected with circTUBGCP3 siRNAs, protein levels of HK1, HK2, pyruvate dehydrogenase, and LDHA were significantly decreased; meanwhile, co-transfection with TUBGCP3-230aa effectively rescued the expression of these enzymes (Fig. 6*A*). Conversely, overexpression of circTUBGCP3 led to increased expression of key glycolytic enzymes, including pyruvate dehydrogenase, PKM1, PKM2, and LDHA, as shown by immunoblotting in AGS cells(Fig. S5, *A*–*C*). We also carried out biochemical glycolysis assays to examine the capacity of circTUBGCP3 and TUBGCP3-230aa to regulate glycolysis *via* ENO1. Lactic acid assays showed that knockdown of circTUBGCP3 significantly reduced lactic acid production in MKN45 cells (Fig. 6*B*, left panel), whereas overexpression of circTUBGCP3 or TUBGCP3-230aa markedly enhanced lactic acid levels in AGS cells (Fig. 6*B*, right panel). Similarly, silencing of circTUBGCP3 resulted in a significant decrease in NADH and ATP production (Fig. 6, *C* and *E*, left panel), while overexpression of circTUBGCP3 or TUBGCP3-230aa promoted the levels of these products (Fig. 6, *D* and *E*, right panel). Glucose uptake and pyruvate production were also assessed. Silencing circTUBGCP3 impaired glucose utilization and decreased pyruvate levels (Fig. 6, *F* and *G*), while overexpression of circTUBGCP3 or TUBGCP3-230aa promoted both glucose consumption and pyruvate accumulation (Fig. 6, *F* and *G*). These results indicate that circTUBGCP3 and TUBGCP3-230aa enhance glycolytic flux by upregulating energy-related metabolites and enzymes.

To determine whether TUBGCP3-230aa-induced glycolysis depends on ENO1, we used ENOblock, a non-substrate analog that inhibits ENO1. TUBGCP3-230aa overexpression upregulated HK1, HK2, pyruvate dehydrogenase, and LDHA expression, but treatment with ENOblock significantly reduced these protein levels in AGS cells (Fig. 6*H*).To further confirm ENO1 dependency, we designed siRNAs targeting ENO1(Fig. S6). Both ENO1 siRNA (siENO1) and ENOblock effectively suppressed the expression of glycolysis-related proteins, including HK1, HK2, ENO1, pyruvate dehydrogenase, and LDHA. Notably, overexpression of linear TUBGCP3-230aa partially rescued the expression of these proteins in cells treated with siENO1 or ENOblock (Fig. 6*I*), supporting the functional role of TUBGCP3-230aa in glycolytic regulation *via* ENO1. *In vivo*, we analyzed tumors from three mice each in the siRNA_nc control group and the siRNA_1-treated group. Western blotting showed that glycolysis-associated proteins-including HK1, HK2, PFKP, PKM1/2, pyruvate dehydrogenase, LDHA, and ENO1-were almost downregulated in tumors from the siRNA_1 group. In two of the three mouse samples, PKM1/2 expression was reduced in the circTUBGCP3 siRNA_1 group, while one sample showed increased expression. This variability is likely due to inter-individual differences among the animals(Fig. 6*J*). To further define the correlations between TUBGCP3-230aa, ENO1, and GPX4 *in vivo*, we also performed IHC assays on the tumor tissues. Tumors from mice treated with circTUBGCP3 siRNAs exhibited reduced expression of ENO1 and GPX4, consistent with our *in vitro* results; HE staining confirmed the histological characteristics of the resected tumors (Fig. S7). These data suggest that TUBGCP3-230aa binding to ENO1 activates the glycolysis pathway in GC *in vivo and in vitro.* The ENO1 inhibitor ENOblock might be an effective therapy target of GC.

### TUBGCP3-230aa suppresses ferroptosis in GC *via* GPX4 in GC cells

**Figure 7:**
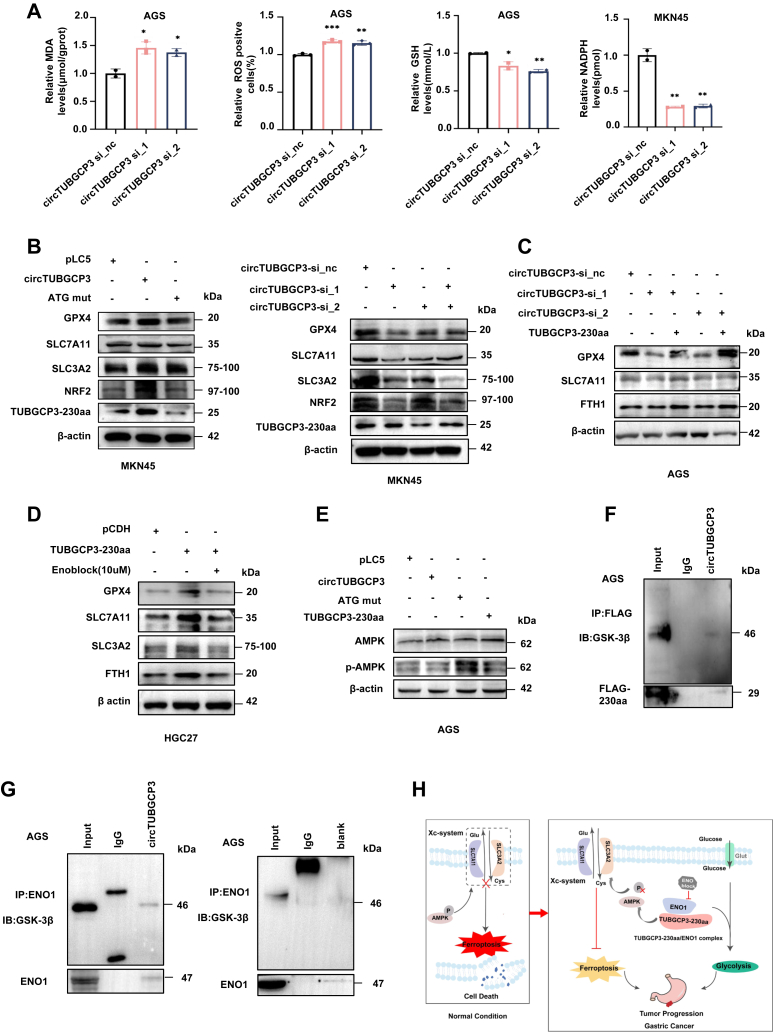
**TUBGCP3-230aa binding ENO1 suppresses ferroptosis *via* the p-AMPK/system Xc-/GPX4 axis in GC cells.***A*, Ferroptosis-related biochemical assays in GC cells. Levels of MDA, ROS, GSH, and NADPH were measured in cells transfected with circTUBGCP3 siRNAs. *B*, immunoblotting to detect key ferroptosis-related proteins in MKN45 cells transfected with circTUBGCP3 overexpression plasmids (*left panel*) or circTUBGCP3 siRNAs (*right panel*). *C*, rescue assays to detect ferroptosis-related proteins in cells co-transfected with circTUBGCP3 siRNAs and linear TUBGCP3-230aa. *D*, rescue assays to detect ferroptosis-related proteins in cells treated with linear TUBGCP3-230aa and the ENO1 inhibitor ENOblock. *E*, immunoblotting to assess AMPK phosphorylation levels following circTUBGCP3 overexpression in MKN45 cells. *F*, Co-IP assays to detect the interaction between TUBGCP3-230aa and GSK3β. *G*, Co-IP assays to determine the interaction between ENO1 and GSK3β in AGS cells with (*left* panel) or without (*right* panel) circTUBGCP3 overexpression. *H*, schematic diagram summarizing the proposed mechanism of this study.

Our IP–MS and KEGG pathway analyses suggested that ferroptosis is one of the key pathways regulated by TUBGCP3-230aa in GC. Moreover, previous studies have shown that ENO1 can function as an RNA-binding protein that degrades mRNA encoding iron regulatory protein 1 (IRP1), thereby influencing ferroptosis and promoting the development of hepatocellular carcinoma (24). Based on these findings, we hypothesized that TUBGCP3-230aa might also regulate ferroptosis *via* ENO1 in GC cells. To test this, we analyzed ferroptosis-associated markers using biochemical assays and immunoblotting. Lipid peroxidation, a hallmark of ferroptosis, was assessed by measuring MDA levels. Inhibition of circTUBGCP3 *via* siRNA significantly increased MDA levels in AGS cells (Fig. 7*A*), while circTUBGCP3 overexpression led to a marked reduction in MDA (Fig. S8). ROS levels were also significantly elevated following circTUBGCP3 knockdown (Figs. 7*A* and S8), indicating enhanced oxidative stress. We next examined GSH, a key intracellular antioxidant. GSH levels were reduced in AGS cells transfected with circTUBGCP3 siRNAs, whereas circTUBGCP3 overexpression led to increased GSH production (Figs. 7*A* and S8). Similarly, NADPH levels, another indicator of antioxidant capacity, were decreased following circTUBGCP3 knockdown in MKN45 cells and increased after overexpression of either circTUBGCP3 or TUBGCP3-230aa in HGC27 cells (Figs. 7*A* and S8). Immunoblotting results confirmed that GPX4, xCT/SLC7A11, SLC3A2, and NRF2 were upregulated in MKN45 cells transfected with circTUBGCP3 plasmids (Fig. 7*B*, left panel), while knockdown of circTUBGCP3 led to their downregulation (Fig. 7*B*, right panel). Additionally, in AGS cells, knockdown of circTUBGCP3 decreased the levels of GPX4, xCT/SLC7A11, and FTH1 proteins. These reductions were partially rescued by co-transfection with TUBGCP3-230aa (Fig. 7*C*). Together, these data indicate that both circTUBGCP3 and TUBGCP3-230aa suppress ferroptosis in GC cells, likely through regulation of the system Xc^-^/GPX4 antioxidant axis.

### Targeting TUBGCP3-230aa influences ferroptosis *via* ENO1/AMPK/GPX4 axis

To further confirm that TUBGCP3-230aa regulates ferroptosis *via* ENO1, we treated GC cells overexpressing TUBGCP3-230aa with the ENO1 inhibitor ENOblock. In HGC27 cells, overexpression of TUBGCP3-230aa increased the expression of key ferroptosis-related proteins, including GPX4, xCT/SLC7A11, SLC3A2, and FTH1. However, this upregulation was markedly suppressed following ENOblock treatment (Fig. 7*D*). To validate these findings *in vivo*, we analyzed xenograft tumor tissues from three mice in each group (siRNA_nc, siRNA_1, and siRNA_2). Western blot analysis revealed that knockdown of circTUBGCP3 decreased the expression of ferroptosis-related proteins (GPX4, xCT/SLC7A11, SLC3A2, NRF2, and FTH1), consistent with the activation of ferroptosis. Most tumor samples from the circTUBGCP3 siRNA groups showed reduced levels of these proteins compared with control tumors. Expression of ENO1 and TUBGCP3-230aa was also decreased in tumors with circTUBGCP3 knockdown (Fig. S9).

AMP-activated protein kinase (AMPK) is known to promote ferroptosis by inhibiting system Xc^-^ activity (25). Given this and our MS data, we hypothesized that AMPK signaling may play a role in TUBGCP3-230aa–mediated ferroptosis suppression. Immunoblotting showed that overexpression of circTUBGCP3 or TUBGCP3-230aa significantly reduced AMPK phosphorylation in AGS cells (Fig. 7*E*), while circTUBGCP3 knockdown led to increased AMPK phosphorylation in MKN45 cells(Fig. S10). These findings suggest that p-AMPK may act as a critical mediator of ferroptosis in the TUBGCP3-230aa/ENO1 regulatory axis. Interestingly, glycogen synthase kinase 3 beta (GSK3β), a known regulator of AMPK activity (26), was among the differentially expressed proteins in our mass spectrometry data. To investigate whether GSK3β is involved in the TUBGCP3-230aa–mediated regulation of AMPK, we carried out Co-IP assays in circTUBGCP3-overexpressing cells using an anti-FLAG antibody. Immunoblotting revealed that TUBGCP3-230aa physically interacts with GSK3β (Fig. 7*F*). Additional IP assays showed that ENO1 also interacted with GSK3β in AGS cells overexpressing circTUBGCP3 (Fig. 7*G*, left panel), but not in cells lacking circTUBGCP3 expression (Fig. 7*G*, right panel). Together, these findings suggest that TUBGCP3-230aa forms a complex with ENO1, which functions as a protein scaffold to recruit GSK3β and modulate AMPK phosphorylation (Fig. 7*H*).

## Discussion

Although the vast majority of circRNAs are classified as non-coding, some have been shown to encode functional peptides (16). To date, the presence of ORFs, m^6^A modifications, and IRESs within circRNA sequences is considered essential for cap-independent translation. For example, circRNAs containing spanning junction ORFs, such as circMbl, can undergo IRES-driven translation (16). Recently, we reported that *circAXIN1* encodes the novel protein AXIN1-295aa, which promotes GC progression (20), and that *circGSPT1* gives rise to GSPT1-238aa, which suppresses GC cell proliferation and invasion (21). These findings highlight the hidden coding potential of circRNAs and underscore their relevance as anticancer targets. In this study, we identified circTUBGCP3, a GC-associated circRNA that encodes a previously undescribed protein, TUBGCP3-230aa. We demonstrated that TUBGCP3-230aa interacts with the glycolytic enzyme ENO1 and promotes tumorigenesis by enhancing glycolysis and suppressing ferroptosis. Molecular assays and xenograft experiments supported the oncogenic role of both circTUBGCP3 and TUBGCP3-230aa, suggesting that this circRNA–protein pair may represent a novel therapeutic target in GC. Moreover, we evaluated the ENO1 inhibitor ENOblock as a potential therapeutic agent for GC and found it could reverse the effects of TUBGCP3-230aa on ferroptosis-associated pathways.

Ferroptosis is a form of regulated cell death characterized by iron-dependent lipid peroxidation, and it plays a crucial role in tumor development (27). The antioxidant system formed by system Xc^-^ (SLC7A11 and SLC3A2) and GPX4 is central to ferroptosis regulation. Downregulation of GPX4 sensitizes cells to ferroptosis, whereas upregulation protects against it. Our findings show that the TUBGCP3-230aa–ENO1 complex suppresses lipid peroxidation *in vitro* and *in vivo*, indicating that TUBGCP3-230aa may be an unexplored regulator in cancer cell ferroptosis. Vasanthi *et al.* (28) showed dependency on GPX4 across diverse cancer therapy-resistant states characterized by high ZEB1 expression levels. System Xc-also has an important role in metastasis, as demonstrated by the suppression of metastasis in a melanoma model following the disruption of xCT (the substrate-specific subunit of system Xc-, also known as SLC7A11) (29). Moreover, Michael *et al.* (30) found that the deletion of Slc7a11 induced tumor-selective ferroptosis and inhibited pancreatic ductal adenocarcinoma growth. Together, these studies support the system Xc^-^–GPX4 axis as an attractive target for cancer therapy. AMPK, a key metabolic regulator, has also been shown to promote ferroptosis by inhibiting GSH biosynthesis (25). Our findings suggest that TUBGCP3-230aa suppresses AMPK phosphorylation, thereby reducing ferroptosis in GC. Thus, modulating the system Xc^-^–GPX4–AMPK network presents a promising strategy for GC intervention.

ENO1 catalyzes the conversion of 2-phosphoglycerate to phosphoenolpyruvate in glycolysis and is frequently overexpressed in multiple cancers, including GC, where it correlates with poor survival (31). Although ENO1 is well known for its metabolic role in supporting tumor proliferation, migration, and invasion (32, 33), recent studies have revealed non-canonical “moonlighting” functions. For instance, ENO1 can bind RNA and degrade IRP1 mRNA, thereby maintaining iron homeostasis and protecting cells from ferroptosis (24). ENO1 has also been implicated in choline phospholipid metabolism and tumor cell proliferation (34). Thus, we add that ENO1 regulates tumor progression through ferroptosis, the mechanisms of which warrant further exploration.

*circTUBGCP3*, derived from exons 12 to 17 of the TUBGCP3 gene, encodes a protein uniquely expressed in GC cells. From a translational perspective, the distinctive C-terminal sequence of TUBGCP3-230aa offers a promising target for antibody-based detection or therapy. Optimized delivery systems or small-molecule inhibitors could be developed to interfere with this signaling axis. We envision the development of combination treatments using anti–TUBGCP3-230aa antibodies alongside ENOblock to improve GC outcomes (35). In summary, our discovery of the novel TUBGCP3-230aa/ENO1 signaling axis sheds new light on the molecular crosstalk between glycolysis and ferroptosis in GC. These findings lay the groundwork for further mechanistic studies and offer new avenues for therapeutic development in gastric cancer.

## Conclusion

In this study, we identified that circTUBGCP3 is overexpressed in gastric cancer (GC) and encodes a novel protein, TUBGCP3-230aa. TUBGCP3-230aa exerts distinct biological functions through its interaction with the glycolytic enzyme ENO1. Both *in vitro* and *in vivo* analyses demonstrated that overexpression of circTUBGCP3 or linear TUBGCP3-230aa promotes GC cell proliferation and migration. Mechanistically, the TUBGCP3-230aa–ENO1 complex enhances glycolysis and recruits GSK3β to suppress ferroptosis by inhibiting AMPK phosphorylation, ultimately contributing to tumor progression (Fig. 7*H*). Our findings uncover circTUBGCP3 and TUBGCP3-230aa as potential biomarkers for GC and reveal a novel mechanism by which ferroptosis is regulated in cancer. Moreover, this study highlights a critical moonlighting function of ENO1 in GC and suggests that targeting the TUBGCP3-230aa–ENO1 axis may represent a promising strategy for cancer therapy.

## Experimental procedures

### Clinical human GC specimens and cell lines

The study was carried out by the principles of the Declaration of Helsinki. Human GC and paired adjacent tissue samples were obtained from Shenzhen University Affiliated Hospital. Informed consent was obtained from all patients, and clinical data, along with postoperative follow-up information, were recorded. All procedures were approved by the Institutional Review Board of the School of Medicine at Shenzhen University (Approval No. PN-202300132).

The 293T cell line and GC cell lines AGS and MKN28 were obtained from the American Type Culture Collection. GES-1, NCI–N87, MKN45, and HGC27 cell lines were purchased from the Cell Bank of the Chinese Academy of Sciences. Immortalized gastric epithelial cells HFE145 were kindly provided by Drs. Hassan Ashktorab and Duane Smoot. All cells were cultured in DMEM (HyClone) supplemented with 10% fetal bovine serum (FBS; Gibco) at 37 °C in a humidified incubator with 5% CO_2_.

### Plasmid construction and cell transfection

The circTUBGCP3-3 × FLAG plasmid, linear TUBGCP3-230aa-3 × FLAG plasmid, circ-frame deletion construct (del flanking), and circTUBGCP3-3 × FLAG ATG mutation construct (ATG mut) were generated by gene synthesis. The pLC5-ciR vector containing artificial flanking sequences was used as the backbone, with a 3 × FLAG tag inserted before the stop codon of the putative open reading frame (ORF). The linear TUBGCP3-230aa-3 × FLAG construct was cloned into the CMV-PCDH vector and served as a positive control. Wild-type and mutant internal ribosome entry sites (IRESs; IRES 323–470, IRES 323–397, IRES 397–470, IRES 606–721, IRES 606–673, and IRES 673–721) were cloned into P-Luc2-IRES-Report vectors (Geneseed, Guangzhou, China).

Small interfering RNAs (siRNAs; Table S1) targeting the junction sites (JS) of circTUBGCP3 were synthesized by Tsingke. Additional siRNAs targeting the circTUBGCP3 cyclization site and ENO1 were synthesized by Prime Biology, Ltd. siRNA nc was used as the negative control. Plasmids were transfected using Lipofectamine 3000 (Invitrogen), and siRNAs were transfected using Lipofectamine RNAiMAX (Invitrogen), following the manufacturer's instructions. All transfections were performed in accordance with standard protocols using Lipofectamine 3000 and P3000 (Thermo Fisher Scientific, Inc.). Primers and siRNA sequences are listed in Table S1.

### Bioinformatics analysis

Prediction of translatable circRNAs was performed using circRNADb (http://reprod.njmu.edu.cn/cgi-bin/circrnadb/circRNADb.php). The ORF structure of circTUBGCP3 was predicted using ORFfinder (https://www.ncbi.nlm.nih.gov/orffinder/). The IRES was predicted using IRES Finder (https://github.com/xiaofeng song/IRESfinder).

We used PyMOL (https://soft.kafan58.com/soft/109969.html?bd_vid=1138608884033 7683059) to predict the binding structure between the TUBGCP3-230aa and ENO1. TUBGCP3-230aa protein's three-dimensional structure modeled by SwissModel (https://swissmodel.expasy.org/). Protein–protein docking simulations were predicted with ClusPro (https://cluspro.bu.edu/home.php). ClusPro 2.0 predicts interactions by computationally simulating the binding modes and affinities between two proteins. Using the Fast Fourier Transform (FFT) algorithm, it efficiently explores all possible translational and rotational combinations of the two proteins (receptor and ligand), generating millions of potential binding conformations.

### Compound treatments

ENOblock was dissolved in DMSO to a concentration of 10 mM and stored at −20 °C. For treatment, ENOblock was added to the culture medium (DMEM supplemented with 10% FBS) at a 1:1000 dilution, and cells were incubated at 37 °C for 24 h. For MG132 treatment, AGS cells were transfected with either an empty vector or the circTUBGCP3 overexpression plasmid. After 48 h, 20 μM MG132 (MCE, HY13259) was added to the culture medium and incubated for 6 h. Total protein was then extracted and analyzed by immunoblotting.

### RNA extraction and real-time quantitative PCR (RT-qPCR)

Total RNA was extracted from tissues and cells using TRIzol reagent (Invitrogen; Thermo Fisher Scientific, Inc.) according to the manufacturer’s protocol. Cytoplasmic and nuclear RNA fractions were extracted using the Cytoplasmic & Nuclear RNA Purification Kit (Norgen Biotek, 37400), and linear RNA was digested with RNase R (0.5 U/μg; GEENSEED, R0301) at 37 °C for 15 min. Reverse transcription was performed using the GoScript Reverse Transcription Mix (Promega, USA), and RT-qPCR was carried out using SYBR Green Master Mix (Promega). Primer sequences are listed in Table S1. The 18S rRNA was used as an internal control.

### RNA fluorescence *in situ* hybridization (FISH)

Cy3-labeled oligonucleotide probes complementary to the junction region of circTUBGCP3 were designed (Table S1). AGS and MKN45 cells were seeded into confocal dishes and cultured overnight. Once cells reached 60 to 70% confluence, FISH assays were performed using the Ribo Fluorescent In Situ Hybridization Kit (Ribobio, R11060.8), following the manufacturer’s protocol. Fluorescent images were acquired using a ZEISS LSM 880 confocal microscope (Carl Zeiss Microscopy GmbH).

### Dual luciferase assay

Predicted IRES sequences and truncated variants were inserted into the P-Luc2-IRES-Report vector (Geneseed). 293T cells were transfected with either the empty vector, wild-type IRES constructs, or deletion (Del) vectors. Luciferase activity was measured using the Dual-Glo Luciferase Assay System (Promega). Both firefly and Renilla luciferase activities were quantified using a microplate reader (TECAN).

### *In vitro* proliferation, migration, and colony formation assays

Cell proliferation was assessed using EdU incorporation assays. Cells were seeded into confocal dishes, transfected, and incubated for 48 h, followed by incubation with 10 μM EdU for 2 h. Cells were then fixed in 4% paraformaldehyde (in PBS) and stained using an EdU assay kit (Ribobio, RN11053.9). Fluorescent images were acquired using a ZEISS LSM 880 confocal microscope.

Wound healing assays were used to evaluate cell migration. Cells were scratched with 10 μl pipette tips and photographed at 0, 24, and 48 h. Migration distance was measured using ImageJ software. For each condition, five random fields were quantified.

Transwell migration assays were also performed. A total of 3 × 104 cells were seeded into the upper chamber of a transwell insert (8 μm pore size; Corning) in medium containing 2% FBS, while the lower chamber contained DMEM with 20% FBS. After 24 h of incubation at 37 °C, migrated cells on the underside of the insert were fixed and stained with hematoxylin. Cells in five random fields per insert were counted under a microscope.

Colony formation assays were used to assess long-term proliferative capacity. Cells transfected with plasmids or siRNAs were seeded in 6-well plates at 1000 cells per well. After 14 days, colonies were fixed with 4% paraformaldehyde, stained with crystal violet, and counted using ImageJ software.

### *In vivo* tumorigenicity assays

Five-week-old female BALB/c nude mice were obtained from Charles River Laboratories. All animal experiments were approved by the Institutional Animal Care and Use Committee of Shenzhen University (Approval No. IACUC-202300140). To investigate the tumorigenic effects of circTUBGCP3 *in vivo*, MKN45 xenograft models were established. Mice were randomly divided into three groups: siRNA_nc, siRNA_1, and siRNA_2. In each group, MKN45 cells (1 × 106 cells in 100 μl PBS) were injected subcutaneously into the abdominal flank region.

One week later, cholesterol-conjugated siRNAs were injected peritumorally twice per week for 3 weeks. For the initial treatment, 10 nmol of cholesterol-conjugated siRNA was administered per tumor every 3 days, followed by weekly treatments until the end of the experiment. Each group consisted of five mice. Tumor volume was measured every 3 days, starting 5 days after cell implantation. At the endpoint, tumors were excised for further analysis.

### Western blotting

A rabbit polyclonal antibody targeting the unique C-terminal peptide (VFCSIRSNS) of TUBGCP3-230aa was custom-generated by AIVD Biotech Corporation. The following primary antibodies were used: anti-ENO1 (Cell Signaling Technology; cat. no. 3810; 1:1000), anti-β-actin (Proteintech, Wuhan, China; cat. no. 81115-1-RR; 1:2000), anti-GAPDH (Cell Signaling Technology, cat. no. 5174; 1:1000), anti-FLAG (Sigma, F1804; 1:1000), anti-ubiquitin (Cell Signaling Technology, cat. no. 3936s; 1:1000), anti-AMPK (Cell Signaling Technology, cat. no. 5831; 1:1000), anti-phospho-AMPK (Cell Signaling Technology, cat. no. 2535; 1:1000), anti-GSK3β (Cell Signaling Technology, cat. no. 12456; 1:1000), Glycolysis Antibody Sampler Kit (Cell Signaling Technology, cat. no. 8337; 1:1000), anti-PKM1 (Proteintech, cat. no. 15821-1-AP; 1:2000), anti-PKM2 (Proteintech, cat. no. 15822-1-AP; 1:2000), and Ferroptosis Antibody Sampler Kit (Cell Signaling Technology, cat. no. 29650; 1:1000).

Cell lysates were prepared using 2 × Laemmli sample buffer (Bio-Rad) supplemented with protease and phosphatase inhibitors (Roche). For tumor tissue samples, proteins were extracted using a tissue protein extraction solution (Sangon). Western blotting was performed as previously described (21).

### Immunoprecipitation and mass spectrometry analysis (IP-MS)

Cells were lysed on ice at 4 °C in ice-cold co-IP lysis buffer containing 25 mM Tris-HCl (pH 7.6), 150 mM NaCl, 0.5% NP-40, 2% PEG, and protease and phosphatase inhibitors. Lysates were cleared by centrifugation at 14,000×*g* for 10 min. Approximately 500 to 1000 μg of total protein was incubated with the immunoprecipitation (IP) antibody at 4 °C overnight. The next day, antigen–antibody complexes were incubated with 25 μl of Pierce agarose magnetic beads A/G (Thermo Fisher Scientific, cat. no. 88804) at room temperature for 2 h. Bound proteins were eluted using 100 μl elution buffer and subsequently analyzed by SDS-PAGE or mass spectrometry (Biotech-Pek). To minimize interference from immunoglobulin heavy and light chains, VeriBlot for IP Detection Reagent (HRP; Abcam, ab131366) was used for detection.

#### Quantification of lactate, ATP, pyruvate, glucose, and NADH

Cells were seeded in six-well plates and transfected with plasmids or siRNAs for 48 h. After harvesting, cells were lysed, and levels of lactate, ATP, pyruvate, glucose, and NADH were measured in the cell extracts using commercially available assay kits: Lactate Assay Kit, ATP Assay Kit, and NADH Assay Kit (NJJC Bio, China); Glucose Assay Kit (Elascience, E-BC-K234-M); and Pyruvate Assay Kit (Solarbio, BC2205, China). All procedures were carried out according to the manufacturer’s instructions.

### NADPH assay

NADPH levels were measured using the NADP^+^/NADPH Assay Kit (Beyotime, S0179). GC cells (1 × 106) were lysed in NADPH extraction buffer, and the lysates were centrifuged. Supernatants were collected and analyzed according to the manufacturer’s instructions.

### Reactive oxygen species (ROS) assay

Intracellular ROS levels were measured using a ROS fluorometric assay kit (Elascience, E-BC-K138-F), following the manufacturer’s instructions. Briefly, GC cells were cultured according to the experimental design. After establishing positive and negative controls, cells were incubated with the fluorescent probe 2′,7′-dichlorofluorescin diacetate (DCFH-DA). Once inside the cell, DCFH-DA is hydrolyzed by intracellular esterases to non-fluorescent DCFH. In the presence of ROS, DCFH is oxidized to DCF, which emits green fluorescence (excitation: 502 nm; emission: 530 nm). The intensity of fluorescence is proportional to the level of intracellular ROS. After incubation with DCFH-DA, cells were resuspended and analyzed according to the kit protocol.

### GSH assay

Reduced glutathione (GSH) levels in GC cells were quantified using a GSH assay kit (Elascience, E-BC-K030). In this assay, GSH reacts with dithiodinitrobenzoic acid (DTNB) to form a yellow-colored product, which was measured spectrophotometrically at 405 nm.

### Lipid peroxidation assay

Lipid peroxidation was assessed by measuring malondialdehyde (MDA) levels using a lipid peroxidation assay kit (Elascience, E-BC-K028-M). MDA reacts with 2-thiobarbituric acid to form reddish-brown chromogenic adducts, which were quantified by measuring absorbance at 532 nm.

### Hematoxylin and eosin (HE) staining & immunohistochemistry (IHC)

To examine tumor tissue morphology in control and treated groups, HE staining was performed on mouse tissue sections. Sections were first immersed in Harris hematoxylin solution for 3 to 8 min, rinsed with tap water, and differentiated in 1% hydrochloric acid alcohol for a few seconds. They were then rinsed again and returned to blue using 0.6% ammonia water, followed by a final rinse under running water. Subsequently, sections were stained with eosin for 1 to 3 min, dehydrated through a graded ethanol series, cleared, and sealed with neutral resin.

Immunohistochemistry was performed to evaluate ENO1 and GPX4 protein expression. Paraffin-embedded tissue sections were dewaxed in xylene and rehydrated through an alcohol gradient. Antigen retrieval was carried out in 10 mM citrate buffer (pH 6.0) using a pressure cooker for 15 min. Endogenous peroxidase activity was blocked with 3% hydrogen peroxide for 10 min. After washing, sections were blocked with 5% BSA and incubated overnight at 4 °C with anti-ENO1 and anti-GPX4 primary antibodies. The following day, sections were incubated with a secondary antibody at 37 °C for 20 min. Color development was performed with freshly prepared DAB solution (MXB, DAB-2031) for 10 min. Slides were counterstained with hematoxylin, dehydrated, cleared, and sealed with neutral resin.

### Statistical analysis

Statistical analyses were conducted using GraphPad Prism 7.0. Comparisons between two groups were performed using the Student’s *t* test, while one-way analysis of variance (ANOVA) was used for comparisons involving more than two groups. Data are presented as means ± standard deviation (SD) from at least three independent experiments. A *p*-value < 0.05 was considered statistically significant.

## Ethics approval

All human tissues were collected from the Department of General Surgery at the first Affiliated Hospital of Shenzhen University with written informed consent. The study was approved by the Clinical Research Ethics Committee (Approval No. PN-202300132). All experimental protocols concerning the handling of mice were approved by the Institutional Animal Care and Use Committee of Shenzhen University (Approval No. IACUC-202300140).

## Data availability

Other data that support the findings of this study are available from the corresponding author upon reasonable request.

## Supporting information

This article contains supporting information.

## Conflict of interest

The authors declare that they do not have any conflicts of interest with the content of this article.
