# Supplementary material for: A novel protein encoded by circTUBGCP3 blocks ferroptosis and promotes gastric cancer progression
Source: J Biol Chem. 2025 Jul 21;301(9):110507. doi: 10.1016/j.jbc.2025.110507 (PMC12926052; doi:10.1016/j.jbc.2025.110507)
Supplement: Supplementary Figures [file mmc1.pdf]

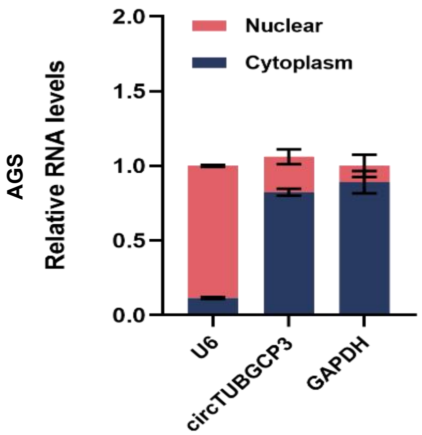

Supplementary Figure 1. Cytoplasmic and nuclear fractions were isolated from AGS, and RT-qPCR was performed to detect the expression of linear TUBGCP3 and circTUBGCP3. GAPDH and U6 RNA served as cytoplasmic and nuclear RNA markers.

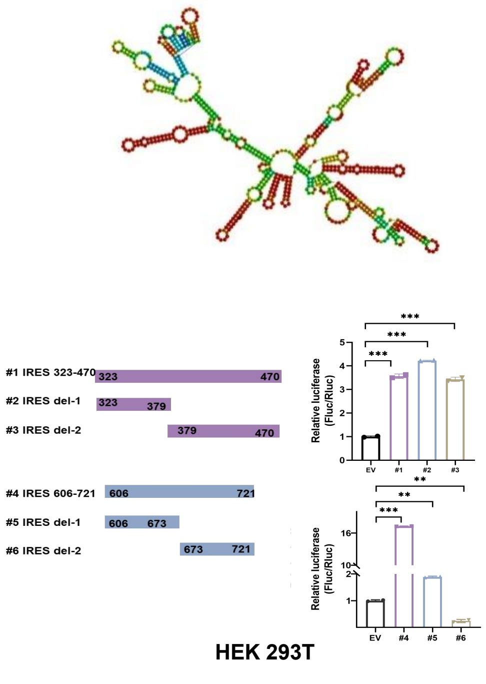

Supplementary Figure 2. Illustration of putative IRESs in circTUBGCP3. Dual luciferase reporter assays were performed to measure IRES activity following different treatments (**P < 0.01; ***p < 0.001).

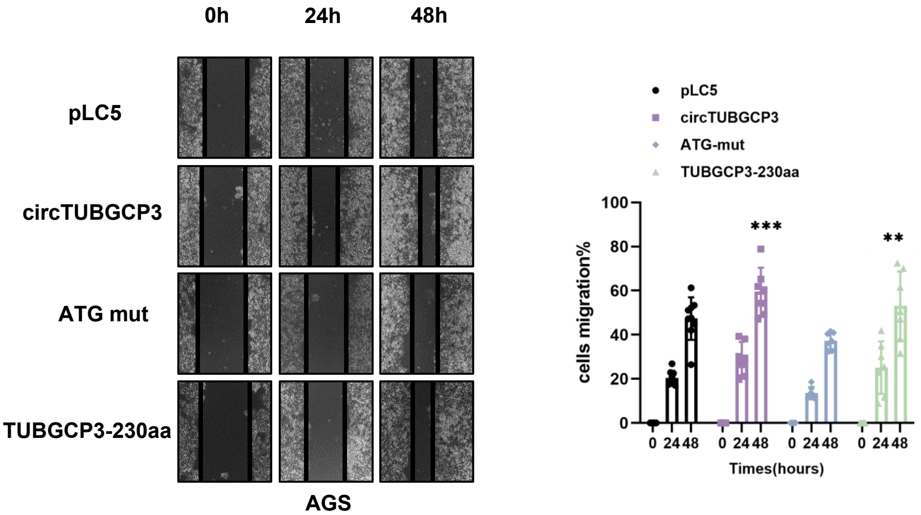

Supplementary Figure 3. Biological functions of circTUBGCP3 in AGS. Wound healing assays were performed to analyze the proliferation and immigration of AGS cells transfected with empty vector and circTUBGCP3 or TUBGCP3-230aa.

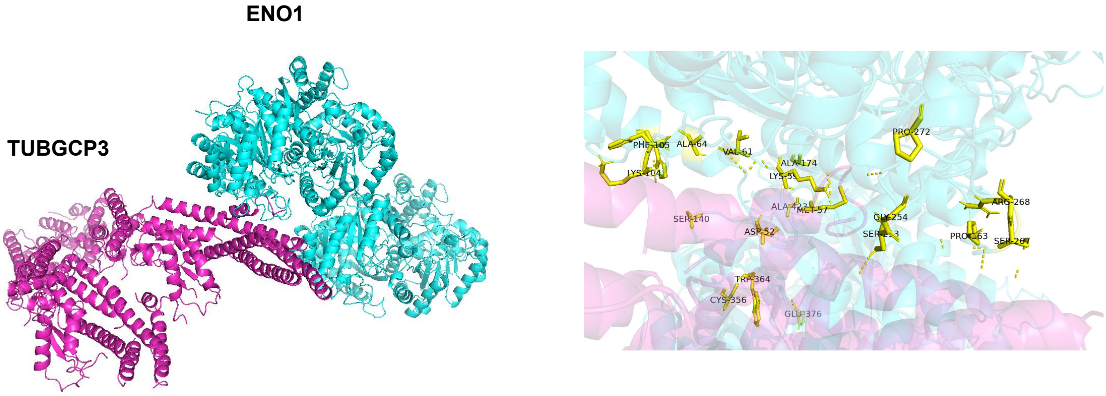

Supplementary Figure 4. ClusPro 2.0 andPyMOL were used to predict the binding modes between the TUBGCP3 and ENO1.

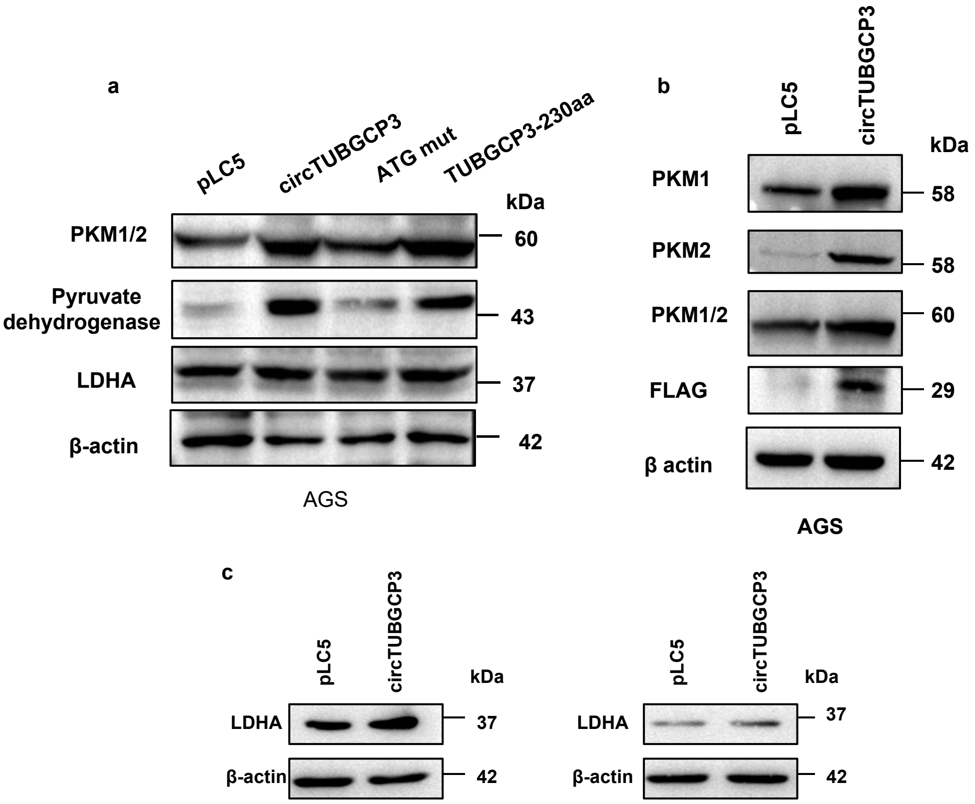

Supplementary Figure 5. Immunoblot to detect key glycolysis enzymes in cells transfected with empty vector, circTUBGCP3 or circTUBGCP3-mut.

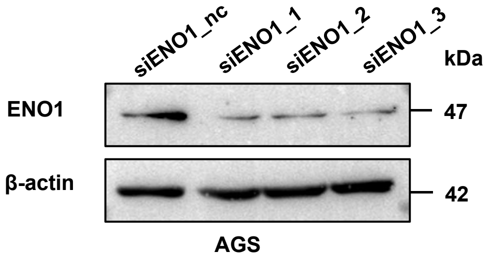

Supplementary Figure 6. Immunoblot to detect ENO1 in AGS transfected with ENO1 siRNAs.

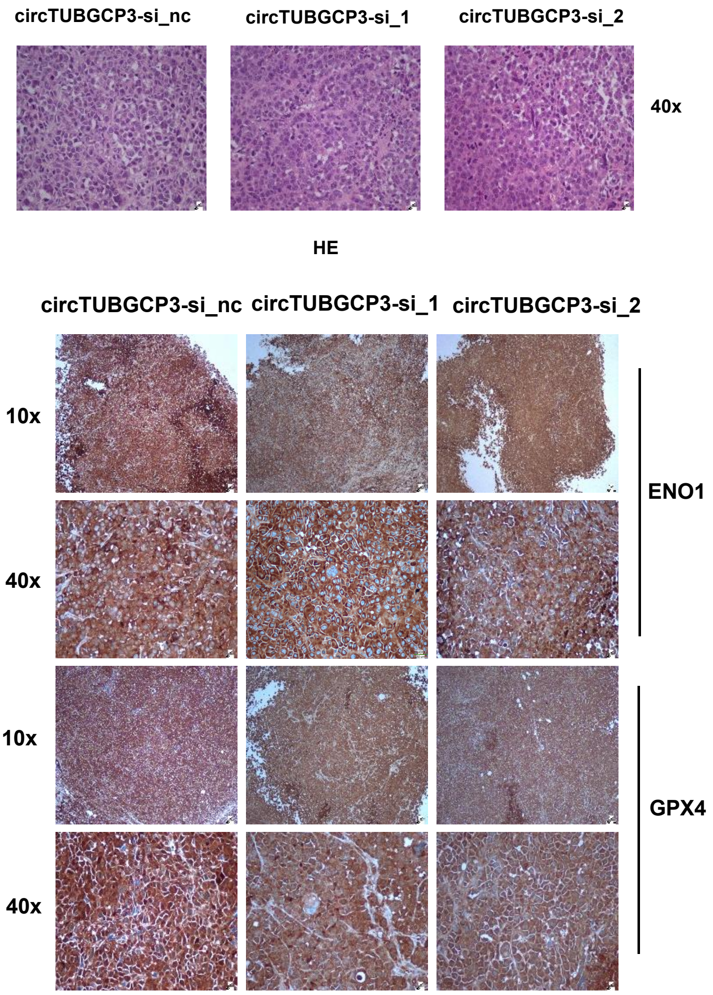

Supplementary Figure 7. Subcutaneous tumor xenograft assays. HE-stained tumor sections from mice in each treatment group, showing subcutaneous tumor morphology. Thelower panel shows representative IHC on tumor sections from mice treated with circTUBGCP3 siRNAs; ENO1 and GPX4 protein levels were detected in nude mouse tumor tissues.

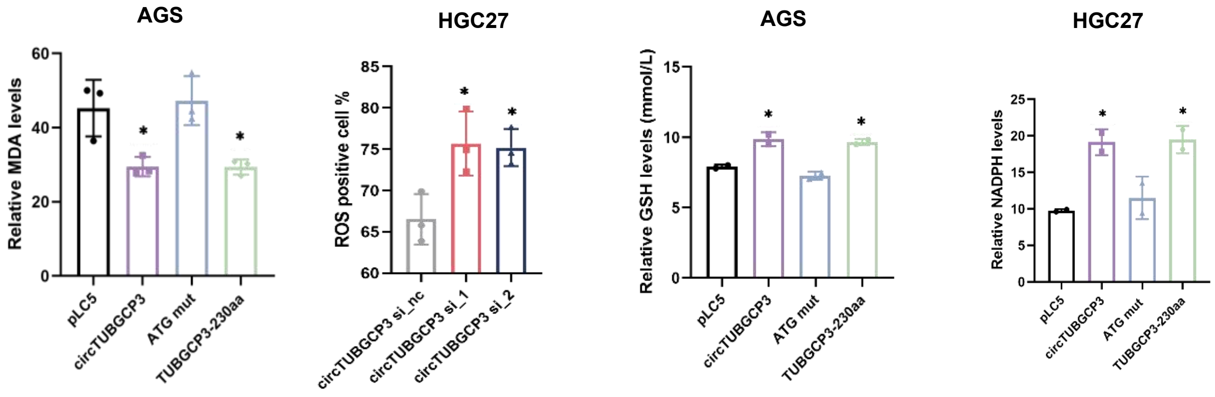

Supplementary Figure 8. Ferroptosis-related biochemical assays in GC cells.The production of MDA, ROS, GSH and NADPH was measured in GC cells with different circTUBGCP3 treatment.

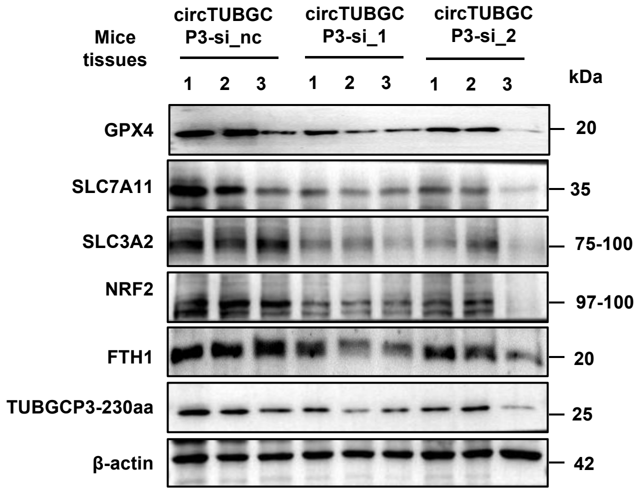

Supplementary Figure 9. The expression levels of ferroptosis-associated proteins were detected in tumor tissues from nude mice treated with ci rcTUBGCP3 siRNAs.

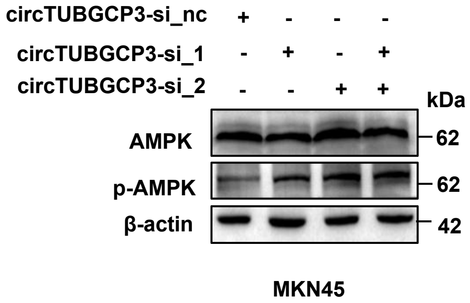

Supplementary Figure 10. The phosphorylation levels of AMPK mediated by GSK-3β. Immunoblotting to detect the AMPK phosphorylation levels by knockdown circTUBGCP3 in MKN45 cells.
