# Supplementary material for: A novel protein encoded by circTUBGCP3 blocks ferroptosis and promotes gastric cancer progression
Source: J Biol Chem. 2025 Jul 21;301(9):110507. doi: 10.1016/j.jbc.2025.110507 (PMC12926052; doi:10.1016/j.jbc.2025.110507)
Supplement: Supplementary Table 1 [file mmc2.docx]

| Supplementary table 1. Sequences of siRNAs，primers and probe | | |
| --- | --- | --- |
| Types | Names | Sequences |
| SiRNAs | hsa_circTUBGCP3_si_nc | ACGUGACACGUUCGGAGAA |
|  | hsa_circTUBGCP3_si_1 | GCUACAAAAAACUGGCAUGUU |
|  | hsa_circTUBGCP3_si_2 | TGCUACAAAAAACUGGCAUGU |
|  | hsa_circTUBGCP3_si_3 | UACAAAAAACUGGCAUGUUUC |
|  | ENO1_si_1 | GAGCAGAGGUUUACCACAA |
|  | ENO1_si_2 | CGAGAUGGAUGGAACAGAA |
|  | ENO1_si_3 | CAGUGGUGUCUAUCGAAGA |
| Primers | qPCR-circTUBGCP3-F | TACGGAAGGGACACATGTGC |
|  | qPCR-circTUBGCP3-R | TACTTGTCGTGCCACAGTCG |
|  | qPCR-TUBGCP3-F | AGGTCTTTGAGAGACACAGCAG |
|  | qPCR-TUBGCP3-R | CAAAAGCTCTGCCCGACGAG |
|  | qPCR-ENO1-F | ATGTCTATTCTCAAGATCCATGCCAGG |
|  | qPCR-ENO1-R | CTTGGCCAAGGGGTTTCTGAAG |
|  | qPCR-hsa_18s-F | ATCCTCAGTGAGTTCTCCCG |
|  | qPCR-hsa_18s-R | CTTTGCCATCACTGCCATTA |
|  | qPCR-hsa_b-actin-F | ACAGAGCCTCGCCTTTGCCGAT |
|  | qPCR-hsa_b-actin-R | CTTGCACATGCCGGAGCCGTT |
| Plasmid construction primers | circTUBGCP3-F | CTTCTTTCGAATTCTAATACTTTCAGTTTTTTGT |
|  | circTUBGCP3-R | AGCTAGGATCCAGTTGTTCTTACCTGGCATGTTT |
|  | circTUBGCP3-ΔFL-R | GGAGTTGTTAGCTAGGATCCCTGGCATGTTTCTC |
|  | linear TUBGCP3-230aa-F | TAGAGCTAGCGAATTCATGATTCCTTCGTTTATG |
|  | linear TUBGCP3-230aa-R | TCGCGGCCGCGGATCCTTACTTGTCATCGTCATC |
| Probes | hsa_circTUBGCP3 | AACATGCCAGTTTTTTGTAGC |
